# Determinants of stillbirths among women who gave birth at Hawassa university comprehensive specialized hospital, Hawassa, Sidama, Ethiopia 2019: a case-control study

**DOI:** 10.1186/s40748-021-00128-4

**Published:** 2021-02-17

**Authors:** Rekiku Fikre, Samuel Ejeta, Taye Gari, Akalewold Alemayhu

**Affiliations:** 1 Mettu University, College of Health Science, P.O. Box 2156, Mettu, Ethiopia; 2 Hawassa University, College of Medicine and Health Science, School of public health, P.O. Box 1560, Awassa, Ethiopia

**Keywords:** Still birth, Determinants, Case-control, Ethiopia

## Abstract

**Background:**

Globally over 2.6 million pregnancy ends with stillbirth annually. Despite this fact, only a few sherds of evidence were available about factors associated with stillbirth in Ethiopia. Therefore, the study aimed to spot factors related to stillbirth among women who gave birth at Hawassa University Comprehensive Specialized Hospital Hawassa, Sidama Ethiopia, 2019.

**Methods:**

Facility-based unmatched case-control study was conducted at Hawassa University Comprehensive Specialized Hospital. Cases were selected using simple random sampling technique and controls were recruited to the study consecutively after every case selection with case to control ratio of 1 to 3. Data were coded and entered into Epi-data version 3.1 and exported to SPSS version 24 for analysis.

**Results:**

A total of 106 cases and 318 controls were included in the study. Number of antenatal care visit [AOR = 0.38, 95% CI (0.15, 0.95)], lack of partograph utilization [AOR = 4.1 95% CI (2.04, 10.5)], prolonged labor [AOR = 6.5, 95% CI (2.9, 14.4)], obstructed labor [AOR = 3.5, 95% CI (1.5, 9.4)], and congenital defect [AOR = 9.7, 95% CI (4.08, 23.0)] were significantly associated with stillbirth.

**Conclusion:**

Absence of partograph utilization, prolonged labor, obstructed labor, antepartum hemorrhage and congenital anomaly were found to have positive association with stillbirth.

## Background

Stillbirth is one of the public health problems and defined as a fetus born with no sign of life at or after 28 completed weeks of gestation with a birth weight of 1000 g or more and a body length of > 35 cm [1–3]. It is an indication for the quality of obstetric care, accessibility as well feasibility of the health care facility to access the service and as a result, it ensued due to the previous history of stillbirth, fetal factor, maternal factors, and poor service delivery associated delay to receive care [2–4].

Globally more than 2.6 million pregnancy ends with stillbirth per annum and the majority of them ensues in the middle and lower-income countries and 55% of this occurs during the intrapartum period [2, 3, 5]. Sub-Saharan and southeast Asian countries account for the highest share of stillbirth, 20 to 40 per 1000 births respectively, which is 10-fold higher compared to developed regions. Evidence suggested that in Finland 2 per 1000 total births verses 40 per 1000 birth in Ni Pakistan, and 30per 1000 in Ethiopia, clearly proved the discrepancy and the standard of maternal care service [6, 7]. Despite stillbirth accounts for huge percent of perinatal mortality, the focus to alleviating the problem is poor as evidence by no emphasis given in the sustainable development goals 2015 [5, 8–10]. The impact of Stillbirth is multi-dimensional. It causes psychological depression for women and it has also effect on the daily life of a family and health care provider [11–15].

Ethiopia showed a decline in maternal mortality by 50% from 1990 to 2016 and neonatal mortality from 55 per 1000 to 28 per 1000 live births between 1990 to 2015 [10]. This progress was due to the expansion of health facilities, an increment of competent providers and improvement in technologies [6, 16].

Despite this fact, Ethiopia ranked 5th in stillbirth rate among the top ten developing countries with 30 per 1000 live births. Approximately over 97,000 still births occur annually and 258 stillbirths occur daily [6].

Therefore, understanding the causes of stillbirth will be important to get timely representative data and run with every newborn action plan targeted to reduce preventable stillbirth to lower than 12 per 1000 live births [6]. The region is one of the three regions with high burden stillbirth next to Oromia and Amhara region [17]. Therefore, this study aimed to investigate key predictors of stillbirth related to some socio-demographic, obstetrics, and medical, fetal and some of the health care factors.

## Methods

### Study setting and period

The study was conducted in Hawassa University comprehensive specialized Hospital Hawassa, Sidama, Ethiopia from June 1, to December 30, 2019. The hospital was located in Sidama Regional State Hawassa city. Hawassa city is found 275 km away from Addis Ababa, the capital of Ethiopia. The hospital was established by the Federal government of Ethiopia in 2005. The hospital was the only teaching and referral hospital in Hawassa city. It has more than 300 beds and It serves for a catchment population of 10–12 million. The hospital gives complete health care delivery services for about 53,384 patients with 8 special departments. Moreover, it provides complete labor and delivery service for the patient visiting from different zones of the region and neighboring regions. Pediatric, obstetrics and gynecology department are the pioneer in the provision of specialized maternal and newborn care including obstetric intensive care unit. In this hospital obstetrics and gynecology department activities have been run with 8 gynecologists, 93 midwives, and with several other staff.

### Population

The source population were all mothers who gave birth at Hawassa University Compressive Specialized Referral Hospital. Cases were all mothers who gave stillbirth at Hawassa University Compressive Specialized Hospital during the period June 1, 2016, to May 31, 2019. Controls were all mothers who gave live birth at Hawassa University Compressive Specialized Hospital during the period June 1, 2016, to May 31, 2019.

### Eligibility criteria

Inclusion criteria for both cases and controls

#### Case

A mother who gave birth at Hawassa university comprehensive specialized hospital during the period June 1, 2016, to May 31, 2019, in maternity ward based on WHO (IDC-10) by a skilled health professional and who had confirmed stillbirth.

#### Control

A mother who gave birth at Hawassa university comprehensive specialized hospital during the period June 1, 2016, to May 31, 2019, in maternity ward based on WHO (IDC-10) by a skilled health professional and who had documented live birth.

### Sample size determination

The Sample size for this study was determined considering factors which have statistically significant association with still birth from previously undertaken studies. Previous studies showed not attending antenatal care, antenatal risk, length of labor, and uterine rupture were factors having statistically significant association with stillbirth. Sample size was estimated by considering case exposed, percent of control exposed, and AOR for each variable. Among the factors used to estimate sample size, not attending antenatal care has given the maximum sample which is 424 (106 cases and 318 controls) below; later on not attending antenatal care is selected by considering maximum sample size (Welegebriel, Dadi et al. 2017) by considering the following assumptions sample size computed by Epi-info version 3.03.

- 95% confidence levels
- 80% power of study
- 1 to 3 cases to control ratio
- AOR = 2.09
- percent of control exposed = 19.9
- percent case exposed = 34.2

Therefore, the final sample size will be recruited in the study = 424 (106 cases and 318 controls) sample size computation

**Table:** 

Variables	Percent of case exposed	Percent of controls exposed	AOR	Sample size			
				Case	Control	Total	References
Uterine rupture	68	22.9	4.9	21	63	84	(Welegebriel, Dadi et al. 2017) [18]
Birth weight < 2.5 kg	17.3	7.1	2.8	98	294	392	(Bayou and Berhan 2012) [19]
Not attending ANC	34.2	19.9	2.09	106	318	424	(Welegebriel, Dadi et al. 2017) [18]
Length of labour> 24 h	43.8	21.3	2.44	49	145	194	(Welegebriel, Dadi et al. 2017) [18]

### Sampling procedure

Hawassa University Comprehensive Specialized Hospital was selected purposefully based on referral status. All mothers who gave birth at Hawassa University Comprehensive Specialized Hospital during the period June 1, 2016, to May 31, 2019, registered in the registration logbook was used source population. There was a total of 15,235 deliveries counted in the logbook over 3 years. From these 316 total stillbirths and 14,919 live births were identified.

The maternal chart that had incomplete documentation were excluded from the study. Finally, 106 cases and 318 controls were selected by using simple random sampling technique by considering the delivery registration book serial number as a sampling frame.

### Operational definition

#### Stillbirth

It is a fetus born dead after 28 weeks of gestational age with a birth weight of > 1000 g during the antepartum or intrapartum period (WHO, 2016).

### Data collection

A pretested and structured checklist that was developed after relevant literature review for the problem under the study was used. The checklist was designed to obtain information that encompasses the main variable for some demographic, obstetrics, and medical complication as well as fetal and health-related factors. Four BSc midwife’s data collectors and one MSc clinical midwifery supervisor were recruited for the data collection. Data collectors and supervisors identify cases and controls along with their respective chart numbers. All selected maternal charts were obtained through communicating with a responsible person of the hospital after a list of cases and controls given to them for trace.

Data collectors review maternal medical chart of case and control from prenatal history and obstetric history, intrapartum follow-up sheet, delivery summary, and laboratory and sonographic notes in the maternal chart were filled into the checklist.

### Data quality control

Two-days training was given for data collectors and supervisors before data collection. Before the actual data collection begin checklist has been pre-tested on 5% of the total sample size at Yirgalem general Hospital. After pretesting questions were revised and adjusted. Data collectors were daily supervised by the supervisor and daily activities were reported to the principal investigator.

### Data analysis

After data collection completed, a filled checklist has been checked for completeness, coded, cleansed then entered into Epi-data 3.1 version. Afterward, it was exported into the statistical package for social science software version 24 for analysis. Descriptive statistics; proportion and cross-tabulation were done to see characteristics of exposure variable between stillbirths and live birth. Variables in the bivariate logistic regression analysis with a *p*-value < 0.25 were included in multivariate logistic regression analysis. Multi collinearity was diagnosed by using the variance inflation factor to see the correlation among independent variables. The Goodness of fit of the final model was checked using Hosmer and Lemeshow test of goodness of fit test. Finally, *p*-value, Adjusted Odds Ratio with 95% CI was presented with narration and table form. Variables having a *p*-value < 0.05 in multivariate logistic regression model were considered to be statistically significant predictors of still birth.

## Results

### Sociodemographic characteristics

**Fig. 1:**
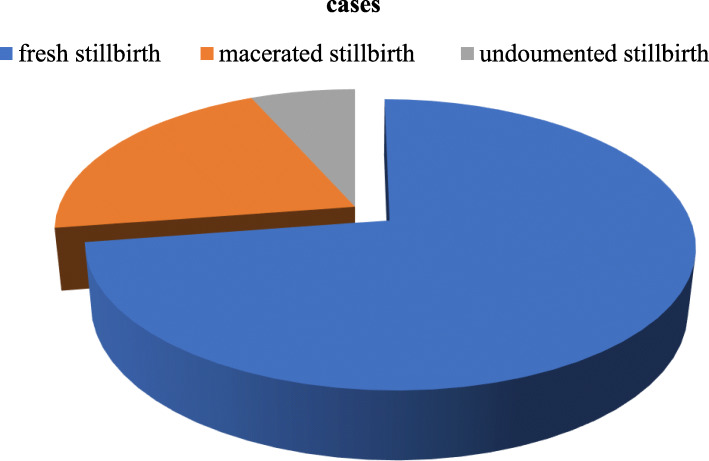
Type of stillbirth among women who gave birth in Hawassa University comprehensive specialized Hospital Hawassa, sidama, Ethiopia 2019

A total of 106 cases and 318 controls women were included in the study. The mean age of the cases was 27.87 with SD + 6.45 and the mean age of the controls was 27.69 with SD + 6.27. Almost half 51(48.1%) of the cases and nearly half 158(47.7%) of controls were ranged in the age group of 21 to 25 years old age. In this study 56(52.8%) of the cases and 130(40.9%) of the controls were from a rural area. Regarding marital status, 101(95.3%) of the cases and 312(98.1%) of the controls were married. However, the pregnancy status of the participant, 79 (74.5%) of the case, and 237(74.5%) of the controls were multigravida mother. (Table 1). Regarding types of stillbirth identified in this study among 106 of the cases 77(72.6%) was a fresh stillbirth, 22(20.8%) was macerated stillbirth and 7(6.6%) of stillbirth unclassified type of stillbirth (Fig. 1).

**Table 1:** Socio-demographic and reproductive characteristics of study Participants in Hawassa University Comprehensive Specialized Hospital, Hawassa, Sidama, 2019

Variable	Category	Cases N (%)	Controls N (%)	*P*-value
Maternal age at birth	16–20	17 (16.0)	54 (17.0)	0.460
	21–25	51 (48.1)	158 (49.7)	
	26–30	13 (12.3)	35 (11)	
	31–35	14 (13.2)	40 (12.6)	
	36–40	11 (10.4)	31 (9.7)	
Residence	Urban	50 (47.2)	188 (59.1)	0.032
	Rural	56 (52.8)	130 (40.9)	
Marital status	Not married	5 (4.7)	6 (1.9)	0.012
	Married	101 (95.3)	312 (98.1)	
Gravidity	primi gravida	27 (25.5)	81 (25.5)	0.077
	Multigravida	79 (74.5)	237 (74.5)	
Parity	Primiparous	54 (50.9)	173 (54.4)	0.325
	Multiparous	29 (27.4)	96 (30.2)	
	GMP	23 (21.7)	49 (15.4)	
Abortion	Yes	32 (30.2)	59 (18.6)	0.012
	No	74 (69.8)	259 (81.4)	
Previous Stillbirth	Yes	12 (11.3)	22 (6.9)	0.148
	No	94 (88.7)	296 (93.1)	

Note: *N* Number while, (%) Represents percent, *MP* Grand multiparous

### Health care service-related characteristics

The majority of the study participants 89(84.0%) of the cases and 264(83.0%) of the controls had antenatal care. More than two thirds, 68(64.2%) of the cases and 98(30.8%) of the controls were referred from other health facilities. Regarding intrapartum labor monitoring, 86(81.1%) of the cases and 298(93.7%) of the controls partograph was utilized.

### Obstetrics and medical illness-related characteristics

Regarding obstetrics complication majority, 92(86.8%) of the cases and 194(61.0%) of the controls had obstetrics complications during pregnancy and delivery. Concerning the onset of labor 66(62.3%) of the cases and 227(71.4%) of the controls labor started spontaneously, while 40(37.7%) of the cases and 91(28.6%) of the controls were labor onset by induction. Nearly three-fourth, 73(68.9%) of the cases and almost half, 156(49.1%) of the controls gave birth vaginally, while 33(31.1%) of the cases and 162(50.9%) of the controls were given birth by caesarian section. Almost more than one thirds 40(37.7%) of the cases and 27(8.5%) of the controls were given birth after 24 h. Nearly half of the study participant 49(46.2%) of the cases and 88(27.2%) of the controls labor started at a health facility. Twenty-two (24.6%) of the cases and 34(10.7%) of the controls had obstructed labor. Although, 7(6.6%) of the cases and 21(6.6%) of the controls had oligo-hydramnios, while 4(3.8%) of the cases and 5(1.6%) of the control had polyhydramnios. Concerning medical illness 53(50.0%) of the cases and 139(43.0%) of controls have a diagnosis of medical complications (Table 2).

**Table 2:** Obstetrics and medical illness-related factors of the study participants of Hawassa University comprehensive Specialized Referral Hospital, Hawassa Sidama, 2019

Variables	Category	Cases N (%)	Controls N (%)	*p*-value
Labor onset	Spontaneous	66 (62.3)	227 (71.4)	0.078
	Induced	40 (37.7)	91 (28.6)	
Mode of delivery	V/D	73 (68.9)	156 (49.1)	0.003
	C/S	33 (31.1)	162 (50.9)	
Length of labour	< 24 h	66 (62.3)	291 (91.5)	0.0001
	> 24 h	40 (37.7)	27 (8.5)	
Where at labor started	At home	57 (53.8)	230 (72.3)	0.0004
	At facility	49 (46.2)	88 (27.7)	
Obstructed labour	Yes	24 (22.6)	34 (10.7)	0.002
	No	82 (77.4)	284 (89.3)	
Oligo-hydramnios	Yes	7 (6.6)	21 (6.6)	1.000
	No	99 (93.4)	297 (93.4)	
Poly-hydramnios	Yes	4 (3.8)	5 (1.6)	0.173
	No	102 (96.2)	313 (98.4)	
multiple pregnancy	Yes	4 (3.8)	12 (3.8)	1.000
	No	102 (96.2)	306 (96.2)	
APH	Yes	40 (37.7)	56 (17.6)	0.0001
	No	66 (62.3)	262 (82.4)	
PROM	Yes	32 (30.2)	85 (26.7)	0.490
	No	74 (69.8)	233 (73.3)	
Chorioamnionitis	Yes	14 (13.2)	31 (9.7)	0.137
	No	92 (86.8)	287 (90.3)	
PIHTN	Yes	29 (27.4)	58 (18.2)	0.044
	No	77 (72.6)	260 (81.8)	
Anemia	Yes	33 (31.1)	50 (15.7)	0.001
	No	73 (68.9)	268 (84.3)	
GDM	Yes	8 (7.5)	13 (4.1)	0.155
	No	98 (92.5)	305 (95.9)	
HIV/AIDS	R	9 (8.5)	38 (11.9)	0.326
	NR	97 (91.5)	280 (88.1)	
Syphilis status	Positive	5 (4.7)	26 (8.2)	0.236
	Negative	101 (95.3)	292 (91.8)	
UTI	Yes	12 (11.3)	35 (11.0)	0.929
	No	94 (88.7)	283 (89.0)	
Malaria	Yes	4 (3.8)	16 (5.0)	0.597
	No	102 (96.2)	302 (95.0)	

Notes: *APH* Antepartum hemorrhage, *PROM* (Premature rupture of membranes), *PIHTN* (pregnancy-induced hypertension), *GHTN* (Gestational hypertension, chronic hypertension) *N* = number while (%) represents percentages, *GDM* Gestational diabetes mellitus

### Fetal related characteristics

On the issue of fetal related characteristics, 68(64.2%) of the cases, and 94(29.6%) of the controls had fetal distress. However, 10(9.4%) of the cases and 9(2.8%) of the controls were have a diagnosis of cord prolapsed. The majority of the study participant, 51(48.1%) of the cases, and 195(61.3%) of the controls were term fetuses. More than two thirds, 61(57.5%) of the cases and 235(73.9%) of the controls had given normal birth weight fetus. About fetal presentation, 73(68.9%) of the cases and 203(63.8%) of the controls were a cephalic presentation during labor and delivery. More than half, 59(55.7%) of the cases and 188(59.1%) of the controls gave birth to the female sex. Furthermore, about the fetal congenital anomalies 21(19.8%) of the cases and 16(5.0%) of the controls had birth defects (Table 3).

**Table 3:** Fetal related factors of study participants in Hawassa University comprehensive Specialized referral Hospital, Hawassa Sidama 2019

Variables	Category	Cases N (%)	Controls N (%)	*P*-value
Fetal status at admission	Normal	38 (35.8)	224 (70.4)	0.0001
	In distress	68 (64.2)	94 (29.6)	
Cord prolapsed	Yes	10 (9.4)	9 (2.8)	0.004
	No	96 (90.6)	309 (97.2)	
Congenital anomalies	Yes	21 (19.8)	16 (5.0)	0.0003
	No	85 (80.2)	302 (95.0)	
Gestational age	< 37 weeks	36 (34.0)	65 (20.4)	0.014
	37 to 42	51 (48.1)	195 (61.3)	
	> 42 weeks	19 (17.9)	58 (18.2)	
Fetal birth weight	LBW	34 (32.1)	52 (16.4)	0.002
	NBW	61 (57.5)	235 (73.9)	
	Macrosomia	11 (10.4)	31 (9.7)	
Fetal presentation	Cephalic	73 (68.9)	203 (63.8)	0.347
	Non-cephalic	33 (31.1)	115 (36.2)	
Fetal sex	Female	59 (55.7)	188 (59.1)	0.532
	Male	47 (44.3)	130 (40.9)	

Notes: *N* Number while, (%) represents a percent. *LBW* Low birth weight, *NBW* Normal birth weight

### Determinants of stillbirth among mother who gave birth at Hawassa comprehensive specialized hospital

The odds of stillbirth is 62% less likely among mothers who had four antenatal care visits compared to mothers who had less than four antenatal care visit [AOR = 0.38 CI (0.15, 0.95)]. However, the odds of women whose birth weren’t followed up by partograph were 4 times higher risk of encountering stillbirth compared to labor monitored by partograph [AOR = 4.1(2.04, 10.5)]. On the other hand, the odd of women who gave birth by cesarean delivery were 67% less likely to have stillbirth than mothers who gave birth through vaginal delivery [AOR = 0.33 CI (0.16, 0.68)]. The odds of a mother who had prolonged labor were 6 times more likely to have stillbirth [AOR = 6.5 CI (2.9, 14.4)] compared to a mother who gave birth within 24 h. Also, the odds of women who had a diagnosis of obstructed labor were 3 times higher risk for [AOR = 3.5 CI (1.5, 9.4)] stillbirth than women who did not have obstructed labor. Moreover, the odds of a mother who had antepartum hemorrhages were 4 times at risk [AOR = 4.3 CI (2.1, 9.05)] for stillbirth than women who did not have antepartum hemorrhages. Finally, odds of women who had a diagnosis of congenital anomalies were 9 times at risk [AOR = 9.7 CI (4.08, 23.0)] for stillbirth than women who did not have congenital anomalies (Table 4).

**Table 4:** logistic regression analysis of factors associated with stillbirth among women who gave birth in Hawassa University comprehensive Specialized referral Hospital, Hawassa, Sidma Ethiopia, 2019

Variable	Category	Case N (%)	Control N (%)	COR 95% CI	AOR 95% CI
Maternal residence	Urban	50 (47.2)	188 (59.1)	1	1
	Rural	56 (52.8)	130 (40.9)	1.62 (1.0,2.52)	1.1 (0.58, 2.13)
Previous stillbirth	Yes	12 (11.3)	22 (6.9)	1.71 (0.81,3.6)	1.6 (0.58, 4.85)
	No	94 (88.7)	296 (93.1)	1	1
ANC visit	1–3 visit	78 (87.6)	196 (74.2)	1	1
	4 visit	11 (12.4)	68 (25.8)	0.40 (0.20,0.81)	0.38 (0.15, 0.95)*
Partograph utilized	Yes	86 (81.1)	298 (93.7)	1	1
	No	20 (18.9)	20 (6.3)	3.46 (1.78,6.73)	4.1 (2.04, 10.5)*
Length o f labour	< 24	66 (62.3)	291 (91.5)	1	1
	> 24	40 (37.7)	27 (8.5)	6.5 (3.7,11.3)	6.5 (2.9, 14.4)***
Mode of delivery	V/D	73 (68.9)	156 (49.1)	1	1
	C/S	33 (31.1)	162 (50.9)	0.43 (0.27,0.69)	0.33 (0.16, 0.68)**
Antepartum hemorrhage	Yes	40 (37.7)	56 (17.6)	6.5 (3.7,11.3)	4.3 (2.1, 9.05)***
	No	66 (62.3)	262 (82.4)	1	1
Pregnancy induced hypertension	Yes	29 (27.4)	58 (18.2)	1.68 (1.01,2.8)	2.08 (0.99, 4.3)
	No	77 (72.6)	260 (81.8)	1	1
Obstructed labour	Yes	24 (22.6)	34 (10.7)	2.44 (1.37,4.35)	3.5 (1.5, 9.4)**
	No	82 (77.4)	284 (89.3)	1	1
Gestational Diabetes Miletus	Yes	8 (7.5)	13 (4.1)	1.91 (0.77,4.75)	2.29 (0.58, 8.9)
	No	98 (92.5)	305 (95.9)	1	1
Anemia	Yes	33 (31.1)	50 (15.7)	2.42 (1.45,4.03)	1.6 (0.79, 3.4)
	No	73 (68.9)	268 (84.3)	1	1
Congenital anomalies	Yes	21 (19.8)	16 (5.0)	4.66 (2.3,9.3)	9.7 (4.08, 23.0) ***
	No	85 (80.2)	302 (95.0)	1	1
Gestational age	Preterm	36 (34.0)	65 (20.4)	1	1
	Term	51 (48.1)	195 (61.3)	0.47 (0.28,0.78)	0.90 (0.35, 2.29)
	Post term	19 (17.9)	58 (18.2)	0.59 (0.30,1.14)	0.75 (0.24, 2.3)
Fetal weight	LBW	34 (32.1)	52 (16.4)	1	1
	NBW	61 (57.5)	235 (73.9)	0.39 (0.23,0.66)	0.53 (0.21, 1.31)
	Macrosomia	11 (10.4)	31 (9.7)	0.54 (0.24,1.22)	0.54 (0.11, 2.6)

Key: reference category = 1: *P*-value significant at: *p < 0.05, **p < 0.01, ***p < 0.001, *N* Number while (%) represents percentages

## Discussion

This study showed that antenatal care visits, lack of partograph utilization and mode of delivery, duration of labor, antepartum hemorrhages, obstructed labor, and congenital anomalies were statistically associated with stillbirth.

The current study showed that odd of a mother who had four antenatal care visits were 62% less likely risk for stillbirth than odds of a mother who have less than four antenatal care visits. This finding was supported by a study conducted in southwest Ethiopia [18], a study in Kenya [20], a study in Nigeria [21], a study in Nepal [22] and study in Zimbabwe [23], a study in Tanzania [24]. The consistency of the finding might be due to methodological similarity between the studies, possibly could be explained antenatal care provide discussion time for women to understanding growth and the requirement of the development for their fetus and the support needed during pregnancy time as well as increase their awareness maintain adequate health. The systematic review revealed antenatal care helps to screen and early detection of pregnancy at risk [4, 25, 26].

This present study also showed that mother her labor not followed by using partograph was 4 times higher at risk for stillbirth than labor followed by partograph. This finding was consistent with a study in Nigeria [21], in Uganda [27], in Nepal [22]. This consistency of the finding could be due to methodological relation between the study and could be explained if partograph not utilized obstructed labor, and prolonged labor which might be led to severe fetal compromise during the intrapartum period difficulty to diagnosis and thus maybe end in stillbirth. The existing evidence also supported that partograph utilization during labor followed and monitoring could be averted stillbirth in a facility where emergency obstetric care is available [1, 28].

This study finds that the odds of mothers gave birth by cesarean delivery was 67% less likely risk of developing stillbirth than odds of mothers gave birth through vaginal delivery.

The finding was consistent with the study in India [29], a study in Tanzania [24]. The comparability of the finding could be explained due to the cesarean section usually done electively based maternal request, in addition to this the exiting data validate caesarian delivery often performed when a vaginal delivery would put a fetus at risk to minimize fetal loss [30]. However, a study conducted in Nepal showed that the mode of delivery not statistically associated with stillbirth [22].

This study also showed odd of women’s diagnosis of prolonged labor was 6 times more at risk for stillbirth than odds of women gave birth within 24 h of the onset of labor. The finding was consistent with the study in Nigeria [31] and study in southwestern Ethiopia [18]. The possible consistency could be due to similarities of methodology between studies and prolonged labor could result in strenuous uterine contraction which maybe results in a non-reassurance fetal heart rate as well as increases the risk of stillbirth outcome. The systematic review and meta-analysis also showed prolonged labor end with stillbirth due to different obstetric difficulties if immediate intervention not undertaken [32, 33]. In a contrary study conducted in Uganda [27], the study in Nigeria [21] duration of labor not shown a statistical association with stillbirth.

In the current study women who had a diagnosis of obstructed labor were 3 times more at risk for stillbirth than women who did not have obstructed labor during labor and delivery. The finding was consistent with a study conducted in south Ethiopia [19] This possible explanation could be due to delay in visiting health facility or referral system, as well as failure to diagnosis and poor emergency preparedness, which results in rupture of the uterus, can be increase stillbirth. In a contrary study in south-west Ethiopia [18] obstructed labor shown no statistical association with stillbirth.

Odd of who had a diagnosis of Antepartum hemorrhage was 4 times more at risk for stillbirth than odds of who women do not have antepartum hemorrhages. This finding was consistent with a study in Nigeria [21], in Zimbabwe [23], and south Ethiopia [18, 19]. The consistency of the findings could be due to methodological similarity of the study, the possible explanation can be some obstetrics causes of antepartum hemorrhage results in excess bleeding which maybe leads anemia and decreased placental perfusion as well as fetal hypoxia that increases the risk of stillbirth. This was confirmed by the available evidence [34].

This study finds the odd women gave fetal congenital anomaly was 9 times at risk for stillbirth than odd women gave fetally had no congenital anomalies. The finding was consistent with a study in Nigeria countries [20, 24]. The possible explanation could be due to methodological familiarity, study population, and the possible explanation could be screening during the antenatal period due to limited advanced diagnostic procedures and related with a skill for prenatal autopsy in developing countries. The systematic review and meta-analysis also reveal that major types of congenital anomaly were incompatible with life [25, 35, 36]. Inconsistently a study conducted in Srilanka [37] (Samaraweera 2019) and Tanzania [24] showed that congenital anomaly no statistical association with stillbirth.

The study reviews multiple exposures for stillbirth as well as both cases and controls were selected from the same setting and population this may reduce selection bias between cases and controls. This study was used secondary data as a source of information, there might be intra-observer bias and diagnosis as well as missing a variable during data collection.

## Conclusion

This study identified antenatal care and caesarian delivery was Negatively associated with stillbirth. Whereas not using partograph, prolonged labor, antepartum hemorrhages, obstructed labor, and congenital anomaly were positively associated with stillbirth. The current study indicated that National policy, structural, and process play a significant role in the quality of service provision. The government plays a role with respective stakeholders to decrease drop out of ANC visits and facilities must give orientation for their providers about respectful and compassionate care and the quality department of respective units must follow the providers during the intrapartum period to follow women with partograph. The study strongly recommended future researchers to do an interventional study that could be addressed preconception care utilization because in current study congenital anomaly the strongest factor associated with stillbirth.

## Data Availability

If you request, we can avail all the data used.

## Abbreviations

APH: Antepartum Hemorrhage
ANC: Antenatal Care
AOR: Adjusted Odd Ratio
WHO: World Health Organization
